# HSV-1 ICP0: An E3 Ubiquitin Ligase That Counteracts Host Intrinsic and Innate Immunity

**DOI:** 10.3390/cells3020438

**Published:** 2014-05-20

**Authors:** Mirna Perusina Lanfranca, Heba H. Mostafa, David J Davido

**Affiliations:** Department of Molecular Biosciences, University of Kansas, 1200 Sunnyside Avenue, Lawrence, KS 66045, USA; E-Mails: mirnapl@ku.edu (M.P.L.); heba@ku.edu (H.H.M.)

**Keywords:** herpes simplex virus, HSV, E3 ubiquitin ligase, infected cell protein 0, ICP0, intrinsic immunity, innate immunity

## Abstract

The herpes simplex virus type 1 (HSV-1) encoded E3 ubiquitin ligase, infected cell protein 0 (ICP0), is required for efficient lytic viral replication and regulates the switch between the lytic and latent states of HSV-1. As an E3 ubiquitin ligase, ICP0 directs the proteasomal degradation of several cellular targets, allowing the virus to counteract different cellular intrinsic and innate immune responses. In this review, we will focus on how ICP0’s E3 ubiquitin ligase activity inactivates the host intrinsic defenses, such as nuclear domain 10 (ND10), SUMO, and the DNA damage response to HSV-1 infection. In addition, we will examine ICP0’s capacity to impair the activation of interferon (innate) regulatory mediators that include IFI16 (IFN γ-inducible protein 16), MyD88 (myeloid differentiation factor 88), and Mal (MyD88 adaptor-like protein). We will also consider how ICP0 allows HSV-1 to evade activation of the NF-κB (nuclear factor kappa B) inflammatory signaling pathway. Finally, ICP0’s paradoxical relationship with USP7 (ubiquitin specific protease 7) and its roles in intrinsic and innate immune responses to HSV-1 infection will be discussed.

## 1. Introduction

Ubiquitination is an important regulator of protein function and stability within cells [1,2]. This system is regulated by an E1-E2-E3 enzymatic cascade that catalyzes the conjugation of ubiquitin (Ub) to target proteins while conferring substrate specificity. Ub monomers are activated by the E1 enzyme, covalently linked to an E2 (of which, at least 35 E2 enzymes are present in humans [3]), and finally transferred from the E2 to the target protein in a mechanism facilitated by one of the many hundreds of E3 Ub ligases [4]. The first ubiquitination event (monoubiquitination) takes place between an internal lysine (K) residue within the target protein and a diglycine motif present in the C-terminus of Ub. As Ub itself contains 7 different lysine residues, differing polyubiquitin branch structures can form depending on which lysine in the initial conjugated Ub moiety is used, which itself is determined by the combined activities of the E2 and E3 enzymes. This conjugation of mono-, multi-, and poly-Ub chains can affect a target protein’s function, localization, and/or stability [5]. Typically, K48 linked Ub chains marks a protein for proteasomal-dependent degradation [6]. In addition, one study showed that ubiquitin was capable of creating linear chains of polyubiquitin through peptide bond linkage to N-terminal amino groups, which alter the functions of target proteins [7]. 

HSV-1 (herpes simplex virus 1) is a ubiquitous human pathogen that infects about 80% of the world population. Infections range from vesicular eruptions around the mouth, called cold sores, to blindness and encephalitis [8]. A hallmark of the infection with HSV-1 is its ability to establish a lifelong latent or quiescent infection in the sensory neurons and to switch from latent to lytic infection when these neurons are stressed [8]. A lytic infection is characterized by the expression of the viral genes in a temporal cascade of immediate early (IE), early (E), and late (L), leading to the production of progeny virus. Latency, on the other hand, is characterized by the lack of infectious virions, while the viral genome persists as an episome in quiescently infected neurons.

Host cells have developed multiple mechanisms to restrict viral infection. Intrinsic immunity is mainly composed of pre-existing proteins that are poised ready to immediately counter the early stages of viral infection [9,10]. This group includes members of nuclear domain 10s (ND10s), which are nuclear proteins important for inhibiting viral replication and repressing viral transcription [11,12,13]. On the other hand, cellular innate immunity is activated by detecting viral components, which establish an antiviral state within the cell. Notably, the type 1 interferon (IFN) response is one of the best studied members of innate immunity [14].

Several viruses including herpesviruses have evolved varied mechanisms to disable the host cell antiviral responses using pathogenicity factors that are structurally and/or functionally similar to E3 Ub ligases [15,16,17,18]. In this review we focus on the HSV-1 E3 Ub ligase, ICP0 (infected cell protein 0), a RING (really interesting new gene)-type E3 ligase that facilitates the transfer of Ub chains to a target substrate by acting as a scaffold that bridges the E2 enzyme and the target protein [19]. We will review ICP0’s ability to target constituents of both the intrinsic and innate branches of the immune system for proteasomal degradation through its E3 Ub ligase activity and the potential benefits or consequences for HSV-1 viral replication. It should be noted that many targets of ICP0 ubiquitination are not exclusively a part of intrinsic or innate defenses but are considered a component of both antiviral responses. For the sake of simplicity, we will primarily describe the role of these cellular factors as either part of intrinsic or innate immunity.

**Figure 1:**
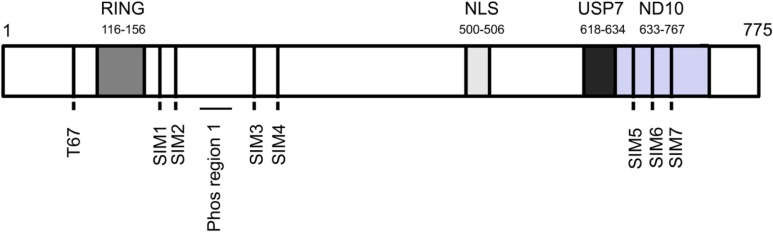
Functional domains and sites in ICP0 relevant to this review. The T67 phosphorylation site, RING-finger motif, ICP0 phosphorylation (Phos) region I (amino acids 224–232), nuclear localization signal (NLS), USP7 binding site, ND10 localization domain, and SIMs (SUMO interaction motifs) 1–7, which start at amino acids 162, 174, 331, 360, 650, 665, and 679 of ICP0, respectively [22].

ICP0 is an IE protein and has been shown to play a key role in regulating the switch between the lytic and latent phases of the viral cycle ([20,21] and reviewed in [22]). Genetic studies using ICP0-null mutants showed that these viruses had impaired growth in cell culture [23], indicating that ICP0 is required for efficient viral replication. The requirement of ICP0 in enhancing viral replication was linked with its ability to transactivate all classes of HSV-1 genes (IE, E, and L) [24]. Due to the presence of a RING-finger motif that mediates the interaction with E2 enzymes (Figure 1), ICP0 was hypothesized to have E3 Ub ligase activity. This hypothesis was confirmed in a series of experiments that demonstrated ICP0’s ability to synthesize chains of poly-Ub *in vitro* and in cell culture [25,26,27,28,29]. ICP0-directed ubiquitination requires one of two known cellular E2 enzymes, UBE2D1 and UBE2E1 [28,29,30,31]. One report showed, using an *in vitro* assay, that ICP0 contained an additional E3 Ub ligase domain in its C-terminus, leading to the ubiquitination and degradation of the E2 protein, cdc34 [32]. However, a later study indicated that viral infection did not alter cdc34 protein levels [33]. Whether cdc34 is a bonafide ICP0 target is unclear, several publications have shown that ICP0 is capable of ubiquitinating a number of targets *in vitro* and in cell culture, including p53 [27] and the ubiquitin specific protease 7 (USP7) [26]. Other reports demonstrated that ICP0 mediates the destruction of the kinetochore proteins, which consequently blocks cell cycle progression and cellular proliferation [34,35,36]. As will be discussed below, the E3 Ub ligase activity of ICP0 plays a central role in HSV-1 replication by impairing components of the hosts’ intrinsic and innate antiviral responses.

## 2. ICP0 Counteracts the Intrinsic Antiviral Resistance of ND10s and SUMO

ND10s are a subnuclear organelle comprised of cellular proteins that are activated by HSV-1 [37] and other DNA- and RNA-containing viruses [13]. ND10s appear to modulate several cellular biological processes, including DNA damage, apoptosis, senescence, IFN response, and protein degradation [13,38,39,40]. Several ND10 constituent proteins, including PML (promyelocytic leukemia), Sp100 (speckled protein of 100 kDa), hDaxx (human death domain-associated protein 6), and ATRX (alpha thalassemia/mental retardation syndrome X-linked) can limit the replication of an ICP0 null HSV-1 mutant [41,42,43]. Upon viral infection, ND10-associated proteins are recruited to the incoming viral genome; however, ICP0’s E3 Ub ligase activity promotes the disruption of ND10 (partially through a C-terminal domain, Figure 1) by mediating the proteolysis (directly or indirectly) of PML and Sp100 [44,45,46,47,48,49,50,51,52] or dissociating hDaxx and ATRX [53] from ND10. The disruption of ND10s by ICP0 alleviates the repressive functions of ND10-associated proteins and stimulates viral transcription. The role of ND10s in the function of ICP0 became apparent when it was shown that depletion of PML, Sp100, hDaxx, or ATRX could partially complement the replication of an ICP0-null mutant but had no effect on wild type HSV-1 [54]. This complementation was further enhanced by the simultaneous depletion of two or more of these proteins [41,54].

ICP0 preferentially directs the degradation of PML and Sp100 that have been modified by one of the small ubiquitin-like modifier (SUMO) proteins [55]. In addition to affecting SUMOylated PML and Sp100, ICP0 reduces the overall level of SUMO-conjugated proteins in the cells, indicating that ICP0 acts as a SUMO targeted Ub ligase (STUbL) [55]. SUMOylation has been shown to repress HSV-1 replication as knock-down of the single SUMO E2 ligase, UBC9, resulted in a complete loss of SUMOylation in cells and enhanced the replication of an ICP0-null mutant [55]. Interestingly, ICP0 contains several SUMO-interacting motifs (SIMs) (Figure 1), likely allowing it to bind to and regulate or ubiquitinate SUMO-modified proteins [55]. Overall, these results suggest that SUMO conjugation and/or SUMO conjugated proteins are part of the host’s intrinsic immune response. A link between PML, Sp100, SUMO, and innate defenses has been suggested as PML and Sp100 are IFN-stimulated genes (ISGs) (reviewed in [39]), with type 1 IFNs being an important part of the innate immune response. Furthermore, SUMOylation of PML and Sp100 increases in IFN-treated cells and correlates with an increase in the size and number of ND10s [56,57]. Overall, ND10s and other SUMOylated proteins appear to recruit cellular antiviral repressors [58], making ND10s and SUMO important players in the intrinsic response against HSV-1, which is ultimately incapacitated by ICP0’s E3 Ub ligase activity.

Besides PML and Sp100, the ND10-associated proteins ATRX and hDaxx promote the formation of repressive chromatin modifications on the incoming HSV-1 genome, though the potential role of ICP0 in counteracting this effect is less well understood. hDaxx can largely function as a transcriptional repressor through the chromatinization of promoters by interacting with histone deacetylases (HDACs) [59,60]. As will be discussed later, ICP0 interacts with and dissociates HDACs, a mechanism hypothesized to be utilized by ICP0 to alleviate the hosts’ transcriptional repression on viral gene expression. Additionally, ATRX and hDaxx were shown to be fundamental regulators of replication-independent chromatin assembly by deposition at telomeres and pericentric heterochromatin [61,62,63]. A recent study using a single cell array with a CMV-promoter-regulated inducible transgene system showed that hDaxx and ATRX expression repressed transcriptional activation, and this was relieved upon expression of ICP0 [64], supporting a role of these factors in silencing viral genomes by an intrinsic antiviral mechanism.

## 3. ICP0 Interferes With the DNA Damage Response, an Intrinsic Defense Against HSV-1

During DNA damage, several signal transduction pathways are initiated with the goal of protecting cells from accumulating or propagating genetic abnormalities. The main signaling pathway mediators that initiate DNA damage response (DDR) include members of the phosphoinositide 3-kinase related kinases (PIKKs): DNA-dependent protein kinase (DNA-PK), ataxia telangectasia mutated (ATM) kinase, and ATM and Rad3 related (ATR) kinase. In addition, the poly (ADP- ribose) polymerase (PARP) family contributes to this signaling. DNA damage by double strand break (DSBs) result in the activation of DNA-PK, ATM, and PARP1/2 [65]; on the other hand, single stranded breaks (SSBs) activate ATR or PARP. Repair of the damaged DNA occurs by homologous recombination or non-homologous end joining repair, and in certain instances, apoptosis can be induced if the damage is extensive [66,67,68]. Typically, signaling through ATR and ATM promotes apoptosis, while DNA-PK leads to non-homologous end-joining repair [69].

Several DNA viruses manipulate the DDR, activating or repressing it to facilitate viral replication (reviewed in [69]). HSV-1 infection has been shown to activate the ATM pathway by inducing the phosphorylation of ATM and its downstream targets [70,71,72]. Additionally, it has been recently reported that HSV-1 infection is associated with activation of PARP1/2. ICP0 has been shown to activate the cellular kinase checkpoint kinase 2 (Chk2), an ATM downstream substrate, blocking the cell cycle in the G2/M phase, an activity which enhances viral replication [73]. On the other hand, DDR activated by HSV-1 is counteracted through the E3 Ub ligase activity of ICP0. Specifically, ICP0 has been shown to direct the proteasomal dependent degradation of the catalytic subunit of DNA-PK (DNA-PKcs) [74,75], which interrupts its repair function and enhances viral replication. Interestingly DNA-PK has been recently shown to activate innate immune mechanisms in response to DNA viruses [76], and this adds potentially another mechanism by which ICP0 counteracts innate immunity.

In addition to DNA-PKcs, ICP0 was shown to ubiquitinate and direct the degradation of RNF8 and RNF168 [77,78]. RNF8 and RNF168 are E3 Ub ligases that act as key mediators of the ATM pathway by poly-ubiquitinating histones, a signal that acts to recruit downstream effectors such as p53BP and BRCA1 to sites of DSBs [79]. Interestingly, extrinsic expression of RNF8 repressed viral transcription [77], and RNF8 degradation is regulated by single site phosphorylation (T67) on ICP0 [80], which facilitates its binding to ICP0. Regarding PARP signaling, ICP0 mediates the proteolysis of poly (ADP-ribose) glycohydrolase (PARG), an enzyme that removes PAR (poly (ADP- ribose)) chains on cellular proteins conjugated by PARP. ICP0 modulation of PARG has been proposed to facilitate viral infection by promoting the PARylation of target proteins, including PARP, involved in the DDR [81]. Overall, ICP0 inhibition of different DNA damage proteins via its E3 Ub ligase enhances viral transcription and replication.

## 4. ICP0’s E3 Ub Ligase Activity Modulates the Activation and Establishment of the IFN Response, an Innate Host Defense

A link between HSV-1 ICP0, and the IFN response was initially established when it was shown that ICP0 mutant viruses replicated poorly in the presence of type I IFNs compared to wild type HSV-1 [82]. In fact, ICP0 was shown to inhibit the induction of IFN-stimulated genes (ISGs) during viral infection [83]. Overall, the expression of ISGs results in the establishment of an antiviral state in cells due to the contribution of the individual activities of many of these interferon stimulated proteins. In fact, ICP0 is known to impair the activation of the type I IFN response through regulatory factors by affecting the levels or activity of many of these proteins amongst which are IFI16, MYD88, and Mal.

### 4.1. IFI16

IFI16, or IFN γ-inducible protein 16, is a DNA sensor that initiates a signaling pathway to induce the expression of type I IFNs. IFI16 was shown to induce IFN-β in response to HSV-1 [84,85] and IFI16’s activation was reportedly linked to its subcellular localization that is strongly linked to its acetylation modification status [86]. The activation of IFI16 upon HSV-1 infection could be counteracted by the expression of ICP0, which promotes its degradation. Further support for a role of IFI16 in counteracting viral replication came from an experiment showing that the depletion of IFI16 partially enhanced the replication of an ICP0-null mutant [84,87]. Interestingly, in addition to its role inducing the IFN response, IFI16 was proposed to silence the HSV-1 genome by triggering heterochromatin association with viral DNA, giving IFI16 an additional function as part of the intrinsic antiviral response. IFI16 promotes the deposition of repressive histone H3K9 trimethylation modifications on HSV chromatin while reducing histone H3K4 trimethylation, which recruits enzymes that add heterochromatin marks for silencing exogenous DNA [88]. This highlights a role of IFI16 in the intrinsic immunity to HSV-1. A recent report, however, has challenged the role of IFI16 in the biology of HSV-1, as the authors of the study presented data reporting that IFI16 degradation was not dependent on ICP0 [87]. Potential explanations regarding the different outcomes between the former and latter studies are that different cell types and conditions were utilized. As examples, the stability of IFI16 may vary in different cell lines, a range of multiplicities of infection were used, and various concentrations of the proteasome inhibitor, MG132, tested could have indirect effects on HSV-1 replication [89].

### 4.2. MyD88 and Mal

Toll like receptors (TLRs) are instrumental in the initial sensing of pathogens and activation of signaling pathways that will ultimately activate the type 1 IFN response [90]. ICP0 was shown to reduce the inflammatory response that TLR2 triggers upon HSV-1 infection [91]. MyD88 (myeloid differentiation factor 88) is an essential adaptor molecule that promotes inflammatory cytokines upon activation of all TLRs. This pathway’s cascade activates the transcription factors NF-κB (nuclear factor kappa B) or AP-1 (activator protein 1) upon initial interaction of MyD88 with the TLRs. Another adaptor molecule necessary in the MyD88-dependent activation of TLR2 and TLR4 is the protein known as Toll/interleukin-1 receptor domain-containing adapter protein (TIRAP) or MyD88 adaptor-like protein (Mal) [92]. ICP0, independent of other viral factors, can block signaling downstream of MyD88 and diminish MyD88 and Mal protein levels [92]. In the latter case, the reduction in MyD88 and Mal protein levels is dependent on the function of ICP0’s E3 Ub ligase activity and the cellular proteasome [91]. These data indicate that ICP0 promotes the degradation of specific TLR adaptor molecules to inhibit the innate inflammatory responses to HSV-1 infection.

### 4.3. NF-κB Signaling

Ubiquitination is essential in the regulation of the tumor necrosis factor (TNF)-α-mediated NF-κB signal transduction pathway [93]. TNF-α is a pro-inflammatory cytokine that is activated upon HSV-1 infection and leads to the induction and regulation of innate and adaptive immune responses through the NF-κB pathway. NF-κB is a family of proteins in which the heterodimer, p65 and p50, represents the predominant form of the transcription factor complex (reviewed in [94]). Several HSV-1 proteins have been shown to perturb NF-κB signaling [95,96,97,98,99]. ICP0 in particular can interact with the NF-κB family members, p65 and p50, and promote the proteasomal degradation of p50; p50 degradation prevents the nuclear translocation of p65, ultimately impeding NF-κB dependent gene expression [100]. In contrast to its NF-κB signaling inhibitory activities, ICP0 has been reported to also ubiquitinate IκBα, consequently stimulating the transcription of NF-κB target genes [101]. Furthermore, NF-κB stimulation can directly benefit HSV-1 replication as it was shown that NF-κB can be recruited to the ICP0 promoter, activating ICP0 transcription and replication [89]. A potential explanation for ICP0’s ability to impair and activate NF-κB signaling is that other HSV-1 proteins might interfere with the antiviral function of specific NF-κB-stimulated proteins; these perturbations are ultimately beneficial for viral replication.

## 5. ICP0 Directs the Degradation of USP7, a Mediator of Intrinsic and Innate Immunity

The ubiquitin specific protease 7 (USP7), also known as herpes-associated ubiquitin specific protease (HAUSP), is a cellular protein that was initially found to strongly interact with the C-terminus of ICP0 [102,103]. USP7 partially colocalizes with ND10, a phenotype that is enhanced in the presence of ICP0 (Figure 1) [104]. Interrupting the interaction between ICP0 and USP7 reduces ICP0’s transactivation activity and viral plaque formation without affecting ND10 disruption [105]. Interestingly, this interaction has been shown to stabilize ICP0 protein levels early during viral infection [106]; however, later during the viral life cycle, ICP0 directs the degradation of USP7, a function that requires ICP0’s RING-finger domain [26] and is regulated by ICP0 phosphorylation [107]. In addition to affecting its stability, ICP0 has been shown to recruit USP7 to the cytoplasm [108]. Cytoplasmic USP7 has been reported to interfere with TLR signaling by the de-ubiquitination of TRAF6 and IKKγ, leading to an inhibition of NF-κB [108]. Another facet of USP7 regulation is that it can interact with and regulate the levels of the E2 enzyme, UbcH6, attenuating its enzymatic functions [109]. Given that UbcH6 interacts with ICP0 to promote the ubiquitination of ICP0 targets PML, p53, and USP7 [26,27,110], it is possible that USP7’s interaction with UbcH6 may modulate ICP0’s E3 Ub ligase activity.

USP7 has been described to have a role in transcriptional repression by stabilizing subunits of the polycomb repressive complex (PRC) [111] and the repressor element 1-silencing transcription factor (REST) [112]. Notably, components of two PRCs have been found to associate with latent HSV-1 genomes [113,114], and ICP0 facilitates HSV-1 reactivation from quiescent infection, which requires its RING-finger motif [115,116]. During reactivation, ICP0 appears to decrease the amount of histones associated with the viral genome while increasing histone acetylation, a modification that typically stimulates transcription [117,118]. These alterations in the chromatinization of the HSV genome during lytic infection and reactivation may also be tied to ICP0’s interactions with and relocalization of histone deacetylases (HDACs), whose activities repress transcription [119]. Interestingly, an ICP0 mutant altered at a series of phosphorylation sites adjacent to the RING-finger (Figure 1), which does not efficiently induce the degradation of USP7 during lytic infection, is impaired for lytic infection and viral reactivation but not latency [107,120]. These results suggests that reductions in USP7 levels mediated by ICP0 may alter chromatinization of the viral genome by destabilizing PRC and REST levels to promote gene expression and reactivation, which is regulated by ICP0 phosphorylation.

An additional aspect of the ICP0/USP7 interaction puzzle is the effect of USP7 degradation on p53 protein levels. p53 has been shown to be a target of ICP0 ubiquitination *in vitro* [27]; however, the levels of p53 are actually stabilized in response to HSV-1 infection. Although the increase in p53 stability can be attributed to USP7 degradation by ICP0, an event which will result in decreased stability of MDM2, an E3 Ub ligase that directs p53 for degradation [121], p53 stabilization in response to HSV-1 infection has been shown to be independent of ICP0 or USP7 [122]. As different stress conditions such as IFN-signaling can result in other post-translational modifications of p53, inducing acetylation, phosphorylation, and SUMOylation, these modifications are critical for p53’s ability to induce apoptosis upon viral infection or senescence in response to IFN treatment [123]. Thus, it is possible that the functional relevance of p53 ubiquitination by ICP0 may be evident only under these circumstances. Indeed ICP0 was shown to inhibit apoptosis when the osteosarcoma cell line, U2OS cells, were exposed to UV irradiation [27]. More studies are required to address the significance of p53 ubiquitination by ICP0 in acute viral infection and reactivation from latency and the effect of USP7 degradation on this process.

## 6. Conclusions

**Figure 2:**
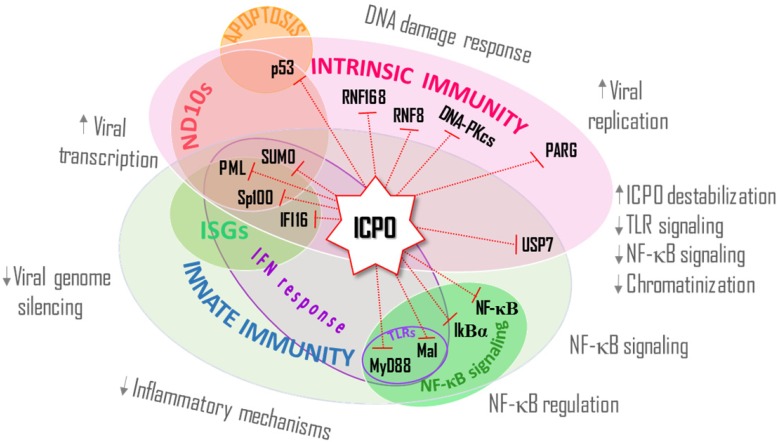
The HSV-1 ICP0 E3 ubiquitin ligase activity counteracts components of host intrinsic and innate immunity by targeting the degradation or dissociation of specific cellular proteins. Many ICP0 targets are multi-functional and participate in both intrinsic and innate antiviral responses and affect several processes, which includes (**1**) Viral transcription: PML, Sp100, and SUMO; (**2**) Viral genome silencing: IFI16; (**3**) Inflammatory mechanisms and NF-κB regulation: IFI16, MyD88, Mal, NF-κB, and IκB; (**4**) Viral replication: PARG; (**5**) DNA damage response: RNF168, RNF8, and DNA-PKcs; and (**6**) ICP0 destabilization, TLR signaling, Chromatinization, NF-κB signaling: USP7.

In order to survive within their host, most viruses have developed several mechanisms to counteract host defenses. The E3 Ub ligase activity of ICP0 is key for HSV-1 evasion of both intrinsic and innate immune responses (Figure 2). This activity of ICP0 is required for efficient viral gene expression and acute viral replication. ICP0 causes the disruption of ND10s, counteracts the DNA damage response, inhibits the IFN pathway, and interferes with cellular transcription repression pathways. Notably, different members of the intrinsic immune response are also considered part of the innate pathway (Figure 2). Interestingly, ICP0 uses different mechanisms to target proteins for degradation or dissociation. For example, the DNA damage protein RNF8 was shown to bind to ICP0 in a phosphorylation dependent manner. On the other hand, several ND10 components including select PML isoforms, Sp100, and hDaxx have not been shown to directly interact with ICP0. This observation indicates that ICP0 can promote the degradation of its targets either directly or indirectly and puts forward the tangible possibility that novel ICP0-cellular binding partners and targets of its degradation will be discovered. Elucidating new targets of ICP0 degradation will help us understand the mechanisms by which ICP0 targets different and interrelated antiviral pathways (e.g., Mal in the IFN response and NF-κB signaling).

In sum, ICP0, through its E3 ubiquitin ligase activity, modulates an array of different and overlapping cellular pathways to ultimately inactivate the cell’s intrinsic and innate antiviral responses, allowing HSV-1 to replicate and persist in its host.

## References

[1] Hershko A., Ciechanover A. (1998). The ubiquitin system. Annu. Rev. Biochem..

[2] Shabek N., Ciechanover A. (2010). Degradation of ubiquitin: The fate of the cellular reaper. Cell Cycle.

[3] Van Wijk S.J., Timmers H.T. (2010). The family of ubiquitin-conjugating enzymes (E2s): Deciding between life and death of proteins. FASEB J..

[4] Metzger M.B., Hristova V.A., Weissman A.M. (2012). HECT and RING finger families of E3 ubiquitin ligases at a glance. J. Cell Sci..

[5] Welchman R.L., Gordon C., Mayer R.J. (2005). Ubiquitin and ubiquitin-like proteins as multifunctional signals. Nat. Rev. Mol. Cell Biol..

[6] Jacobson A.D., Zhang N.Y., Xu P., Han K.J., Noone S., Peng J., Liu C.W. (2009). The lysine 48 and lysine 63 ubiquitin conjugates are processed differently by the 26 s proteasome. J. Biol. Chem..

[7] Tokunaga F., Sakata S., Saeki Y., Satomi Y., Kirisako T., Kamei K., Nakagawa T., Kato M., Murata S., Yamaoka S. (2009). Involvement of linear polyubiquitylation of NEMO in NF-kappaB activation. Nat. Cell Biol..

[8] Roizman R., Whitley R.J., Knipe D.M. (2007). Herpes Simplex Viruses.

[9] Yan N., Chen Z.J. (2012). Intrinsic antiviral immunity. Nat. Immunol..

[10] Bieniasz P.D. (2004). Intrinsic immunity: A front-line defense against viral attack. Nat. Immunol..

[11] Regad T., Saib A., Lallemand-Breitenbach V., Pandolfi P.P., de The H., Chelbi‐Alix M.K. (2001). PML mediates the interferon-induced antiviral state against a complex retrovirus via its association with the viral transactivator. EMBO J..

[12] McNally B.A., Trgovcich J., Maul G.G., Liu Y., Zheng P. (2008). A role for cytoplasmic PML in cellular resistance to viral infection. PLoS One.

[13] Tavalai N., Stamminger T. (2008). New insights into the role of the subnuclear structure ND10 for viral infection. Biochim. Biophys. Acta.

[14] Ivashkiv L.B., Donlin L.T. (2014). Regulation of type I interferon responses. Nat. Rev. Immunol..

[15] Coscoy L., Ganem D. (2000). Kaposi’s sarcoma-associated herpesvirus encodes two proteins that block cell surface display of MHC class I chains by enhancing their endocytosis. Proc. Natl. Acad. Sci. USA.

[16] Ishido S., Choi J.K., Lee B.S., Wang C., DeMaria M., Johnson R.P., Cohen G.B., Jung J.U. (2000). Inhibition of natural killer cell-mediated cytotoxicity by Kaposi’s sarcoma-associated herpesvirus K5 protein. Immunity.

[17] Zhu H., Zheng C., Xing J., Wang S., Li S., Lin R., Mossman K.L. (2011). Varicella-zoster virus immediate-early protein ORF61 abrogates the IRF3-mediated innate immune response through degradation of activated IRF3. J. Virol..

[18] Wang L., Oliver S.L., Sommer M., Rajamani J., Reichelt M., Arvin A.M. (2011). Disruption of PML nuclear bodies is mediated by ORF61 SUMO-interacting motifs and required for varicella-zoster virus pathogenesis in skin. PLoS Pathog..

[19] De Bie P., Ciechanover A. (2011). Ubiquitination of E3 ligases: Self-regulation of the ubiquitin system via proteolytic and non-proteolytic mechanisms. Cell Death Differ..

[20] Leib D.A., Coen D.M., Bogard C.L., Hicks K.A., Yager D.R., Knipe D.M., Tyler K.L., Schaffer P.A. (1989). Immediate-early regulatory gene mutants define different stages in the establishment and reactivation of herpes simplex virus latency. J. Virol..

[21] Cai W.Z., Schaffer P.A. (1989). Herpes simplex virus type 1 ICP0 plays a critical role in the de novo synthesis of infectious virus following transfection of viral DNA. J. Virol..

[22] Boutell C., Everett R.D. (2013). Regulation of alphaherpesvirus infections by the ICP0 family of proteins. J. Gen. Virol..

[23] Sacks W.R., Schaffer P.A. (1987). Deletion mutants in the gene encoding the herpes simplex virus type 1 immediate-early protein ICP0 exhibit impaired growth in cell culture. J. Virol..

[24] Cai W., Schaffer P.A. (1992). Herpes simplex virus type 1 ICP0 regulates expression of immediate-early, early, and late genes in productively infected cells. J. Virol..

[25] Everett R.D. (2000). ICP0 induces the accumulation of colocalizing conjugated ubiquitin. J. Virol..

[26] Boutell C., Canning M., Orr A., Everett R.D. (2005). Reciprocal activities between herpes simplex virus type 1 regulatory protein ICP0, a ubiquitin E3 ligase, and ubiquitin-specific protease USP7. J. Virol..

[27] Boutell C., Everett R.D. (2003). The herpes simplex virus type 1 (HSV-1) regulatory protein ICP0 interacts with and Ubiquitinates p53. J. Biol. Chem..

[28] Boutell C., Sadis S., Everett R.D. (2002). Herpes simplex virus type 1 immediate-early protein ICP0 and is isolated RING finger domain act as ubiquitin E3 ligases *in vitro*. J. Virol..

[29] Hagglund R., Van Sant C., Lopez P., Roizman B. (2002). Herpes simplex virus 1-infected cell protein 0 contains two E3 ubiquitin ligase sites specific for different E2 ubiquitin-conjugating enzymes. Proc. Natl. Acad. Sci. USA.

[30] Gu H., Roizman B. (2003). The degradation of promyelocytic leukemia and Sp100 proteins by herpes simplex virus 1 is mediated by the ubiquitin-conjugating enzyme UbcH5a. Proc. Natl. Acad. Sci. USA.

[31] Vanni E., Gatherer D., Tong L., Everett R.D., Boutell C. (2012). Functional characterization of residues required for the herpes simplex virus 1 E3 ubiquitin ligase ICP0 to interact with the cellular E2 ubiquitin-conjugating enzyme UBE2D1 (UbcH5a). J. Virol..

[32] Hagglund R., Roizman B. (2002). Characterization of the novel E3 ubiquitin ligase encoded in exon 3 of herpes simplex virus-1-infected cell protein 0. Proc. Natl. Acad. Sci. USA.

[33] Everett R.D. (2004). Herpes simplex virus type 1 regulatory protein ICP0 does not protect cyclins D1 and D3 from degradation during infection. J. Virol..

[34] Everett R.D., Earnshaw W.C., Findlay J., Lomonte P. (1999). Specific destruction of kinetochore protein CENP-C and disruption of cell division by herpes simplex virus immediate-early protein Vmw110. EMBO J..

[35] Lomonte P., Sullivan K.F., Everett R.D. (2001). Degradation of nucleosome-associated centromeric histone H3-like protein CENP-A induced by herpes simplex virus type 1 protein ICP0. J. Biol. Chem..

[36] Lomonte P., Morency E. (2007). Centromeric protein CENP-B proteasomal degradation induced by the viral protein ICP0. FEBS Lett..

[37] Everett R.D., Murray J., Orr A., Preston C.M. (2007). Herpes simplex virus type 1 genomes are associated with ND10 nuclear substructures in quiescently infected human fibroblasts. J. Virol..

[38] Everett R.D., Chelbi-Alix M.K. (2007). PML and PML nuclear bodies: Implications in antiviral defence. Biochimie.

[39] Regad T., Chelbi-Alix M.K. (2001). Role and fate of PML nuclear bodies in response to interferon and viral infections. Oncogene.

[40] Bernardi R., Papa A., Pandolfi P.P. (2008). Regulation of apoptosis by PML and the PML-NBs. Oncogene.

[41] Everett R.D., Parada C., Gripon P., Sirma H., Orr A. (2008). Replication of ICP0-null mutant herpes simplex virus type 1 is restricted by both PML and Sp100. J. Virol..

[42] Everett R.D., Rechter S., Papior P., Tavalai N., Stamminger T., Orr A. (2006). PML contributes to a cellular mechanism of repression of herpes simplex virus type 1 infection that is inactivated by ICP0. J. Virol..

[43] Negorev D.G., Vladimirova O.V., Ivanov A., Rauscher F. (2006). Maul GG Differential role of Sp100 isoforms in interferon-mediated repression of herpes simplex virus type 1 immediate-early protein expression. J. Virol..

[44] Muller S., Dejean A. (1999). Viral immediate-early proteins abrogate the modification by SUMO-1 of PML and Sp100 proteins, correlating with nuclear body disruption. J. Virol..

[45] Chelbi-Alix M.K., de, Thé H (1999). Herpes virus induced proteasome-dependent degradation of the nuclear bodies-associated PML and Sp100 proteins. Oncogene.

[46] Everett R.D., Freemont P., Saitoh H., Dasso M., Orr A., Kathoria M., Parkinson J. (1998). The disruption of ND10 during herpes simplex virus infection correlates with the Vmw110- and proteasome-dependent loss of several PML isoforms. J. Virol..

[47] Maul G.G., Everett R.D. (1994). The nuclear location of PML, a cellular member of the C3HC4 zinc-binding domain protein family, is rearranged during herpes simplex virus infection by the C3HC4 viral protein ICP0. J. Gen. Virol..

[48] Everett R.D., Maul G.G. (1994). HSV-1 IE protein Vmw110 causes redistribution of PML. EMBO J..

[49] Everett R.D., Freemont P., Saitoh H., Dasso M., Orr A., Kathoria M., Parkinson J. (1998). The disruption of ND10 during herpes simplex virus infection correlates with the Vmw110- and proteasome-dependent loss of several PML isoforms. J. Virol..

[50] Parkinson J., Everett R.D. (2000). Alphaherpesvirus proteins related to herpes simplex virus type 1 ICP0 affect cellular structures and proteins. J. Virol..

[51] Walters M.S., Kyratsous C.A., Silverstein S.J. (2010). The RING finger domain of Varicella-Zoster virus ORF61p has E3 ubiquitin ligase activity that is essential for efficient autoubiquitination and dispersion of Sp100-containing nuclear bodies. J. Virol..

[52] Lanfranca M.P., Mostafa H.H., Davido D.J. (2013). Two overlapping regions within the N-terminal half of the herpes simplex virus 1 E3 ubiquitin ligase ICP0 facilitate the degradation and dissociation of PML and dissociation of Sp100 from ND10. J. Virol..

[53] Lukashchuk V., Everett R.D. (2010). Regulation of ICP0-null mutant herpes simplex virus type 1 infection by ND10 components ATRX and hDaxx. J. Virol..

[54] Glass M., Everett R.D. (2013). Components of promyelocytic leukemia nuclear bodies (ND10) act cooperatively to repress herpesvirus infection. J. Virol..

[55] Boutell C., Cuchet-Lourenco D., Vanni E., Orr A., Glass M., McFarlane S., Everett R.D. (2011). A viral ubiquitin ligase has substrate preferential SUMO targeted ubiquitin ligase activity that counteracts intrinsic antiviral defence. PLoS Pathog..

[56] Grotzinger T., Sternsdorf T., Jensen K., Will H. (1996). Interferon-modulated expression of genes encoding the nuclear-dot-associated proteins Sp100 and promyelocytic leukemia protein. Eur. J. Biochem..

[57] Guldner H.H., Szostecki C., Grotzinger T., Will H. (1992). IFN enhance expression of Sp100, an autoantigen in primary biliary cirrhosis. J. Immunol..

[58] Cuchet-Lourenco D., Boutell C., Lukashchuk V., Grant K., Sykes A., Murray J., Orr A., Everett R.D. (2011). SUMO pathway dependent recruitment of cellular repressors to herpes simplex virus type 1 genomes. PLoS Pathog..

[59] Hollenbach A.D., McPherson C.J., Mientjes E.J., Iyengar R. (2002). Grosveld G Daxx and histone deacetylase II associate with chromatin through an interaction with core histones and the chromatin-associated protein Dek. J. Cell Sci..

[60] Li R., Pei H., Watson D.K., Papas T.S. (2000). EAP1/Daxx interacts with ETS1 and represses transcriptional activation of ETS1 target genes. Oncogene.

[61] Drane P., Ouararhni K., Depaux A., Shuaib M., Hamiche A. (2010). The death-associated protein DAXX is a novel histone chaperone involved in the replication-independent deposition of H3.3. Genes Dev..

[62] Goldberg A.D., Banaszynski L.A., Noh K.M., Lewis P.W., Elsaesser S.J., Stadler S., Dewell S., Law M., Guo X.Y., Li X. (2010). Dstinct factors control histone variant H3.3 localization at specific genomic regions. Cell.

[63] Lewis P.W., Elsaesser S.J., Noh K.M., Stadler S.C., Allis C.D. (2010). Daxx is an H3.3-specific histone chaperone and cooperates with ATRX in replication-independent chromatin assembly at telomeres. Proc. Natl. Acad. Sci. USA.

[64] Newhart A., Rafalska-Metcalf I.U., Yang T., Negorev D.G. (2012). Janicki SM Single-cell analysis of Daxx and ATRX-dependent transcriptional repression. J. Cell Sci..

[65] Dantzer F., Ame J.C., Schreiber V., Nakamura J., Menissier-de Murcia J, Murcia G. (2006). Poly(ADP-ribose) polymerase-1 activation during DNA damage and repair. Methods Enzymol..

[66] Abraham R.T. (2004). PI 3-kinase related kinases: “Big” players in stress-induced signaling pathways. DNA Repair (Amst).

[67] Ciccia A., Elledge S.J. (2010). The DNA damage response: Making it safe to play with knives. Mol. Cell.

[68] Haince J.F., McDonald D., Rodrigue A., Dery U., Masson J.Y., Hendzel M.J., Poirier G.G. (2008). PARP1-dependent kinetics of recruitment of MRE11 and NBS1 proteins to multiple DNA damage sites. J. Biol. Chem..

[69] Turnell A.S., Grand R.J. (2012). DNA viruses and the cellular DNA-damage response. J. Gen. Virol..

[70] Shirata N., Kudoh A., Daikoku T., Tatsumi Y., Fujita M., Kiyono T., Sugaya Y., Isomura H., Ishizaki K., Tsurumi T. (2005). Activation of ataxia telangiectasia-mutated DNA damage checkpoint signal transduction elicited by herpes simplex virus infection. J. Biol. Chem..

[71] Lilley C.E., Carson C.T., Muotri A.R., Gage F.H., Weitzman M.D. (2005). DNA repair proteins affect the lifecycle of herpes simplex virus 1. Proc. Natl. Acad. Sci. USA.

[72] Wilkinson D.E., Weller S.K. (2004). Recruitment of cellular recombination and repair proteins to sites of herpes simplex virus type 1 DNA replication is dependent on the composition of viral proteins within prereplicative sites and correlates with the induction of the DNA damage response. J. Virol..

[73] Li H., Baskaran R., Krisky D.M., Bein K., Grandi P., Cohen J.B., Glorioso J.C. (2008). Chk2 is required for HSV-1 ICP0-mediated G2/M arrest and enhancement of virus growth. Virology.

[74] Lees-Miller S.P., Long M.C., Kilvert M.A., Lam V., Rice S.A., Spencer C.A. (1996). Attenuation of DNA-dependent protein kinase activity and its catalytic subunit by the herpes simplex virus type 1 transactivator ICP0. J. Virol..

[75] Parkinson J., Lees-Miller S.P., Everett R.D. (1999). Herpes simplex virus type 1 immediate-early protein vmw110 induces the proteasome-dependent degradation of the catalytic subunit of DNA-dependent protein kinase. J. Virol..

[76] Ferguson B.J., Mansur D.S., Peters N.E., Ren H., Smith G.L. (2012). DNA-PK is a DNA sensor for IRF-3-dependent innate immunity. Elife.

[77] Lilley C.E., Chaurushiya M.S., Boutell C., Everett R.D. (2011). Weitzman MD The intrinsic antiviral defense to incoming HSV-1 genomes includes specific DNA repair proteins and is counteracted by the viral protein ICP0. PLoS Pathog..

[78] Lilley C.E., Chaurushiya M.S., Boutell C., Landry S., Suh J., Panier S., Everett R.D., Stewart S.G., Durocher, D. (2010). A viral E3 ligase targets RNF8 and RNF168 to control histone ubiquitination and DNA damage responses. EMBO J..

[79] Bekker-Jensen S., Mailand N. (2010). Assembly and function of DNA double-strand break repair foci in mammalian cells. DNA Repair (Amst).

[80] Chaurushiya M.S., Lilley C.E., Aslanian A., Meisenhelder J., Scott D.C., Landry S., Ticau S., Boutell C., Yates R.Y., Schulman A.B. (2012). Viral E3 ubiquitin ligase-mediated degradation of a cellular E3: Viral mimicry of a cellular phosphorylation mark targets the RNF8 FHA domain. Mol. Cell.

[81] Grady S.L, Hwang J., Vastag L., Rabinowitz J.D., Shenk T. (2012). Herpes simplex virus 1 infection activates poly(ADP-ribose) polymerase and triggers the degradation of poly(ADP-ribose) glycohydrolase. J. Virol..

[82] Mossman K. (2005). Analysis of anti-interferon properties of the herpes simplex virus type I ICP0 protein. Methods Mol. Med..

[83] Eidson K.M., Hobbs W.E., Manning B.J., Carlson P., DeLuca N.A. (2002). Expression of herpes simplex virus ICP0 inhibits the induction of interferon-stimulated genes by viral infection. J. Virol..

[84] Orzalli M.H., DeLuca N.A., Knipe D.M. (2012). Nuclear IFI16 induction of IRF-3 signaling during herpesviral infection and degradation of IFI16 by the viral ICP0 protein. Proc. Natl. Acad. Sci. USA.

[85] Soby S., Laursen R.R., Ostergaard L., Melchjorsen J. (2012). HSV-1-induced chemokine expression via IFI16-dependent and IFI16-independent pathways in human monocyte-derived macrophages. Herpesviridae.

[86] Li T., Diner B.A., Chen J., Cristea I.M. (2012). Acetylation modulates cellular distribution and DNA sensing ability of interferon-inducible protein IFI16. Proc. Natl. Acad. Sci. USA.

[87] Cuchet-Lourenco D., Anderson G., Sloan E., Orr A., Everett R.D. (2013). The viral ubiquitin ligase ICP0 is neither sufficient nor necessary for degradation of the cellular DNA sensor IFI16 during herpes simplex virus 1 infection. J. Virol..

[88] Orzalli M.H., Conwell S.E., Berrios C., DeCaprio J.A., Knipe D.M. (2013). Nuclear interferon-inducible protein 16 promotes silencing of herpesviral and transfected DNA. Proc. Natl. Acad. Sci. USA.

[89] La Frazia S., Amici C., Santoro M.G. (2006). Antiviral activity of proteasome inhibitors in herpes simplex virus-1 infection: Role of nuclear factor-kappaB. Antivir. Ther..

[90] Lester S.N., Li K. (2013). Toll-Like Receptors in Antiviral Innate Immunity. J. Mol. Biol..

[91] Van Lint A.L., Murawski M.R., Goodbody R.E., Severa M., Fitzgerald K.A., Finberg W.R., Knipe M.D., Kurt-Jones A.E. (2010). Herpes simplex virus immediate-early ICP0 protein inhibits Toll-like receptor 2-dependent inflammatory responses and NF-kappaB signaling. J. Virol..

[92] Fitzgerald K.A., Palsson-McDermott E.M., Bowie A.G., Jefferies C.A., Mansell A.S., Brady G, Brint E., Dunne1 A., Gray P. (2001). Mal (MyD88-adapter-like) is required for Toll-like receptor-4 signal transduction. Nature.

[93] Ea C.K., Deng L., Xia Z.P., Pineda G., Chen Z.J. (2006). Activation of IKK by TNFalpha requires site-specific ubiquitination of RIP1 and polyubiquitin binding by NEMO. Mol. Cell.

[94] Hayden M.S., Ghosh S. (2004). Signaling to NF-kappaB. Genes Dev..

[95] Xing J., Ni L., Wang S., Wang K., Lin R., Zheng C. (2013). Herpes simplex virus 1-encoded tegument protein VP16 abrogates the production of beta interferon (IFN) by inhibiting NF-kappaB activation and blocking IFN regulatory factor 3 to recruit its coactivator CBP. J. Virol..

[96] Zhang J., Wang S., Wang K., Zheng C. (2013). Herpes simplex virus 1 DNA polymerase processivity factor UL42 inhibits TNF-alpha-induced NF-kappaB activation by interacting with p65/RelA and p50/NF-kappaB1. Med. MicroBiol. Immunol..

[97] Jin H., Ma Y., Yan Z., Prabhakar B.S., He B. (2012). Activation of NF-kappaB in CD8+ dendritic cells *Ex Vivo* by the gamma134.5 null mutant correlates with immunity against herpes simplex virus 1. J. Virol..

[98] Cotter C.R., Kim W.K., Nguyen M.L., Yount J.S., Lopez C.B., Blaho J.A., Moran T.M. (2011). The virion host shutoff protein of herpes simplex virus 1 blocks the replication-independent activation of NF-kappaB in dendritic cells in the absence of type I interferon signaling. J. Virol..

[99] Kim J.C., Lee S.Y., Kim S.Y., Kim J.K., Kim H.J., Lee HM, Choi M.S., Min J.S., Kim M.J., Choi H.S. (2008). HSV-1 ICP27 suppresses NF-kappaB activity by stabilizing IkappaBalpha. FEBS Lett..

[100] Zhang J., Wang K., Wang S., Zheng C. (2013). Herpes simplex virus 1 E3 ubiquitin ligase ICP0 protein inhibits tumor necrosis factor alpha-induced NF-kappaB activation by interacting with p65/RelA and p50/NF-kappaB1. J. Virol..

[101] Diao L., Zhang B., Fan J., Gao X., Sun S., Yang K., Xin D., Jin N., Geng Y., Wang C. (2005). Herpes virus proteins ICP0 and BICP0 can activate NF-kappaB by catalyzing IkappaBalpha ubiquitination. Cell Signal..

[102] Meredith M., Orr A., Elliott M., Everett R.D. (1995). Separation of sequence requirements for HSV-1 Vmw110 multimerisation and interaction with a 135-kDa cellular protein. Virology.

[103] Meredith M., Orr A., Everett R.D. (1994). Herpes simplex virus type 1 immediate-early protein Vmw110 binds strongly and specifically to a 135-kDa cellular protein. Virology.

[104] Everett R.D., Meredith M., Orr A., Cross A., Kathoria M., Parkinson J. (1997). A novel ubiquitin-specific protease is dynamically associated with the PML nuclear domain and binds to a herpesvirus regulatory protein. EMBO J..

[105] Everett R.D., Meredith M., Orr A. (1999). The ability of herpes simplex virus type 1 immediate-early protein Vmw110 to bind to a ubiquitin-specific protease contributes to its roles in the activation of gene expression and stimulation of virus replication. J. Virol..

[106] Canning M., Boutell C., Parkinson J., Everett R.D. (2004). A RING finger ubiquitin ligase is protected from autocatalyzed ubiquitination and degradation by binding to ubiquitin-specific protease USP7. J. Biol. Chem..

[107] Mostafa H.H., Thompson T.W., Davido D.J. (2013). N-terminal phosphorylation sites of herpes simplex virus type 1 ICP0 differentially regulate its activities and enhance viral replication. J. Virol..

[108] Daubeuf S., Singh D., Tan Y., Liu H., Federoff H.J., Bowers W.J., Tolba K. (2009). HSV ICP0 recruits USP7 to modulate TLR-mediated innate response. Blood.

[109] Sarkari F., Wheaton K., La Delfa A., Mohamed M., Shaikh F., Khatun R., Arrowsmith C.H., Frappier L., Saridakis V., Sheng Y. (2013). Ubiquitin-specific protease 7 is a regulator of ubiquitin-conjugating enzyme UbE2E1. J. Biol. Chem..

[110] Boutell C., Orr A., Everett R.D. (2003). PML residue lysine 160 is required for the degradation of PML induced by herpes simplex virus type 1 regulatory protein ICP0. J. Virol..

[111] De Bie P., Zaaroor-Regev D., Ciechanover A. (2010). Regulation of the Polycomb protein RING1B ubiquitination by USP7. Biochem. Biophys Res. Commun..

[112] Huang Z., Wu Q., Guryanova O.A., Cheng L., Shou W., Rich J.N., Bao S. (2011). Deubiquitylase HAUSP stabilizes REST and promotes maintenance of neural progenitor cells. Nat. Cell Biol..

[113] Kwiatkowski D.L., Thompson H.W., Bloom D.C. (2009). The polycomb group protein Bmi1 binds to the herpes simplex virus 1 latent genome and maintains repressive histone marks during latency. J. Virol..

[114] Cliffe A.R., Coen D.M., Knipe D.M. (2013). Kinetics of facultative heterochromatin and polycomb group protein association with the herpes simplex viral genome during establishment of latent infection. MBio.

[115] Ferenczy M.W., DeLuca N.A. (2011). Reversal of heterochromatic silencing of quiescent herpes simplex virus type 1 by ICP0. J. Virol..

[116] Ferenczy M.W., Ranayhossaini D.J., Deluca N.A. (2011). Activities of ICP0 involved in the reversal of silencing of quiescent herpes simplex virus 1. J. Virol..

[117] Cliffe A.R., Knipe D.M. (2008). Herpes simplex virus ICP0 promotes both histone removal and acetylation on viral DNA during lytic infection. J. Virol..

[118] Coleman H.M., Connor V., Cheng Z.S., Grey F., Preston C.M., Efstathiou S. (2008). Histone modifications associated with herpes simplex virus type 1 genomes during quiescence and following ICP0-mediated de-repression. J. Gen. Virol..

[119] Lomonte P., Thomas J., Texier P., Caron C., Khochbin S., Epstein A.L. (2004). Functional interaction between class II histone deacetylases and ICP0 of herpes simplex virus type 1. J. Virol..

[120] Mostafa H.H., Thompson T.W., Kushnir A.S., Haenchen S.D., Bayless A.M., Hilliard J.G., Link M.A., Pitcher L.A., Loveday E., Schaffer P.A. (2011). Herpes simplex virus 1 ICP0 phosphorylation site mutants are attenuated for viral replication and impaired for explant-induced reactivation. J. Virol..

[121] Li M., Chen D., Shiloh A., Luo J., Nikolaev A.Y., Qin J., Gu W. (2002). Deubiquitination of p53 by HAUSP is an important pathway for p53 stabilization. Nature.

[122] Boutell C., Everett R.D. (2004). Herpes simplex virus type 1 infection induces the stabilization of p53 in a USP7- and ATM-independent manner. J. Virol..

[123] Marcos-Villar L., Perez-Giron J.V., Vilas J.M., Soto A., de la Cruz-Hererra C.F., Lang V., Collado M., Vidal A., Rodríguez M.S., Muñoz-Fontela C. (2013). SUMOylation of p53 mediates interferon activities. Cell Cycle.

